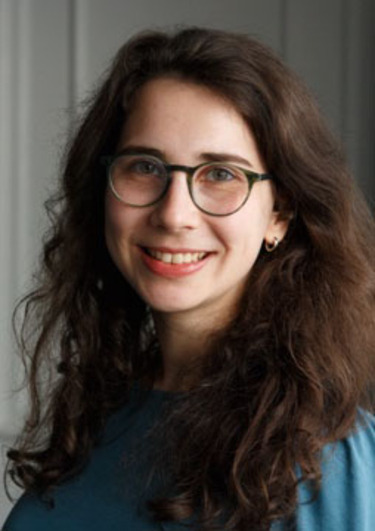# Detecting the Undetectable: Advances in Methods for Identifying Small Tau Aggregates in Neurodegenerative Diseases

**DOI:** 10.1002/cbic.202400877

**Published:** 2025-01-08

**Authors:** Dorothea Böken, Yunzhao Wu, Ziwei Zhang, David Klenerman

**Affiliations:** ^1^ Yusuf Hamied Department of Chemistry University of Cambridge Cambridge CB2 1EW UK; ^2^ UK Dementia Research Institute University of Cambridge Cambridge CB2 0AH UK

**Keywords:** Tau, oligomer, single-molecule

## Abstract

Tau, a microtubule‐associated protein, plays a critical role in maintaining neuronal structure and function. However, in neurodegenerative diseases such as Alzheimer's disease and other tauopathies, tau misfolds and aggregates into oligomers and fibrils, leading to neuronal damage. Tau oligomers are increasingly recognised as the most neurotoxic species, inducing synaptic dysfunction and contributing to disease progression. Detecting these early‐stage aggregates is challenging due to their low concentration and high heterogeneity in biological samples. Traditional methods such as immunostaining and enzyme‐linked immunosorbent assay (ELISA) lack the sensitivity and specificity to reliably detect small tau aggregates. Advanced single‐molecule approaches, including single‐molecule fluorescence resonance energy transfer (smFRET) and single‐molecule pull‐down (SiMPull), offer improved sensitivity for studying tau aggregation at the molecular level. These emerging tools provide critical insights into tau pathology, enabling earlier detection and characterisation of disease‐relevant aggregates, thereby offering potential for the development of targeted therapies and diagnostic approaches for tauopathies.

## Graphical abstract

This review explores current methods for detecting and characterising small tau aggregates, which are increasingly recognised as key drivers of neurotoxicity in tauopathies. It discusses emerging techniques such as single‐molecule pull‐down (SiMPull), single‐molecule Förster resonance energy transfer (smFRET), and cryo‐electron tomography (cryo‐ET), highlighting their potential to improve our understanding of diseases such as Alzheimer's where traditional methods fall short.

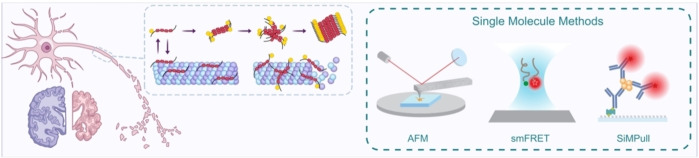

## 1 Introduction

Tau is a microtubule‐associated protein (MAP) that primarily facilitates the stabilisation of microtubules in axons. As such, tau functions as part of the cytoskeleton and is important for maintaining cellular integrity and synaptic connectivity.^[1]^ It is also involved in axonal transport, neurite growth, and nucleic acid protection. However, several factors (such as post‐translational modifications and mutations) can decrease tau's affinity for the microtubules and/or induce tau misfolding, thereby increasing its propensity to aggregate, leading to the formation of oligomers and their subsequent aggregation into insoluble fibrils.[^2^, ^3^] The accumulation of these fibrils results in the formation of intracellular neurofibrillary tangles (NFTs), which are one of the pathological hallmarks of a group of diseases known as tauopathies. These include, for example, Alzheimer's disease (AD), frontotemporal dementia (FTD), corticobasal degeneration (CBD) and progressive supranuclear palsy (PSP). The presence of NFTs is strongly correlated with neurodegeneration, cognitive decline, and other neurological deficits observed in tauopathies, underscoring the importance of tau aggregation in disease progression.

Significant progress has been made in understanding the structural properties of *in vitro* and *ex vivo* tau aggregates. For example, the atomic structures of heparin‐induced tau fibrils and brain‐derived tau filaments have been resolved,[^4^, ^5^] exhibiting distinct characteristics; also indicating the potential presence of non‐proteinaceous cofactors within some fibrils.[^6^, ^7^] However, the underlying mechanisms of tau aggregation remain only partially understood. Tau aggregation, encompassing oligomerisation and fibril formation, is considered a central event in the pathogenesis of AD and other tauopathies, making it a compelling target for drug discovery and development.^[8]^ However, the molecular and cellular factors that initiate tau misfolding and aggregation and drive the spread of tau pathology within the brain remain elusive.

**Scheme 1:**
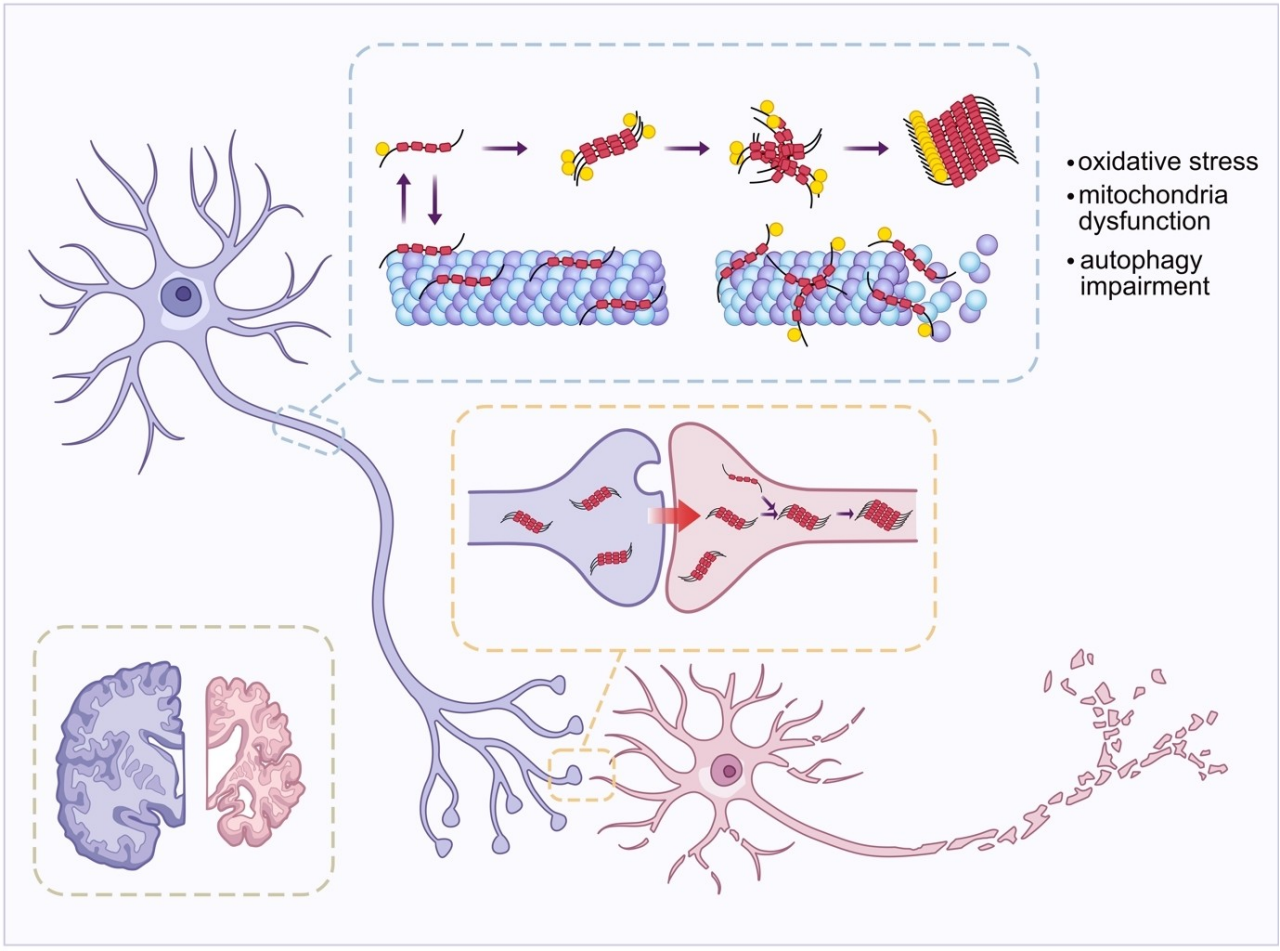
Schematic representation of tau aggregation and propagation in neurodegenerative disease. Tau monomers undergo misfolding, hyperphosphorylation and aggregation into oligomers and protofibrils, which eventually form mature fibrils. These tau aggregates disrupt microtubule stability and induce cellular dysfunction, including oxidative stress, mitochondrial dysfunction, and autophagy impairment. Tau aggregates spread trans‐synaptically between neurons, promoting the propagation of pathology and contributing to neurodegeneration.

More critically, the link between tau aggregation and pathology is a major focus of current research. Unlike other aggregating proteins, tau does not readily aggregate by itself under physiological conditions; aggregation typically requires specific factors, such as post‐translational modifications such as phosphorylation,^[9]^ cofactors such as heparin^[10]^ or truncation.^[11]^ Tau aggregation is a complex and dynamic process involving multiple aggregate species, including oligomers, protofibrils, and fibrils, which are morphologically and functionally distinct; therefore, elucidating the role of different tau aggregates in disease, i. e., which aggregates can induce neuronal damage and ultimately lead to tauopathy, is critical to the development of effective therapeutics (Scheme 1). Increasing evidence suggests that tau oligomers, rather than larger aggregates such as NFTs, are the most neurotoxic species.^[12]^ These oligomers can induce synaptic dysfunction, membrane damage, and cellular stress, leading to neuronal death.[^13^, ^14^, ^15^] In contrast, NFTs, once thought to be the primary driver of neurodegeneration, are now considered to be relatively inert and do not appear to promote further tau aggregation.[^16^, ^17^] Instead, they are increasingly recognised as a potential neuroprotective mechanism that sequesters harmful tau species.[^18^, ^19^]

Furthermore, tauopathies such as AD, FTD, CBD and PSP are characterised by different tau aggregation profiles and exhibit distinct pathological signatures.^[20]^ The correlation between specific tau aggregate species and the clinical manifestations of these diseases remains unclear, but understanding this relationship is crucial for developing targeted therapeutics. In AD, tau aggregates are predominantly composed of paired helical filaments (PHFs) and straight filaments (SFs) formed by a mix of 3R (three microtubule‐binding repeats) and 4R (four microtubule‐binding repeats) tau isoforms.^[4]^ These filaments adopt a characteristic C‐shaped fold in AD. In contrast, tau filaments in PSP and CBD are composed exclusively of 4R tau isoforms and exhibit distinct structural morphologies. PSP filaments form a straight, three‐layered fold,^[20]^ while CBD filaments adopt a four‐layered twisted morphology with unique cross‐β‐sheet arrangements.^[6]^ PSP and CBD also feature characteristic astrocytic pathology, with PSP marked by tufted astrocytes (astrocytic inclusions tau filaments radiating outward in a tuft‐like pattern) and CBD characterised by astrocytic plaques (star‐shaped tau inclusions with a dense central core surrounded by a network of filaments).^[21]^ Pick's disease, a rare form of frontotemporal dementia, presents a distinct pathology marked by the presence of Pick bodies; large, spherical intracellular inclusions primarily composed of 3R tau isoforms. The tau filaments in Pick's disease adopt a unique J‐shaped fold.^[22]^ Pick bodies are predominantly found in the hippocampus and cortical regions, and their presence correlates with the severe neuronal loss and cognitive decline characteristic of Pick's disease.^[23]^

These differences in tau structures and aggregation patterns may explain differences in disease progression and symptoms[^20^, ^24^] and may present opportunities for developing targeted therapies aimed at specific tau isoforms or aggregate morphologies.

Additionally, tau aggregation leads to the depletion of functional, tau, which is essential for maintaining neuronal health by stabilising microtubules and supporting cellular transport.[^25^, ^26^] It remains unclear how much of the pathology is directly caused by the toxicity of tau aggregates versus the loss of these physiological functions. This balance between the gain of toxic tau species and the loss of normal tau function is critical for understanding how aggregation drives neurodegeneration.

Despite the progress made in this field, our understanding of tau aggregation is hampered by significant methodological limitations.

One of the major challenges is the inability to accurately detect and measure small tau aggregates or oligomers, which are believed to be highly toxic intermediates in the aggregation process that induce synaptic loss and dysfunction, mitochondrial impairment, and inflammation.[^27^, ^28^, ^29^, ^30^] This challenge is twofold: on the one hand, these aggregates are small, ranging from tens to hundreds of nanometres in size depending on the aggregation stage,[^31^, ^32^, ^33^] and typically present at low concentrations (nM~pM),[^15^, ^32^] which places high demands on the sensitivity of the detection method. Current techniques, such as immunohistochemistry and various imaging methods, are not sensitive enough to reliably detect these early‐stage tau species *in vivo* or in post‐mortem human tissue. On the other hand, methods with high spatial resolution and/or high sensitivity, such as electron microscopy, are often hindered by the lack of specificity for these small aggregates, especially in complex samples such as brain tissue.

Many experimental approaches used to study tau aggregation also oversimplify the process and do not fully capture the complexity of tau's behaviour *in vivo*. For instance, *in vitro* studies often rely on truncated tau fragments or artificial aggregation inducers such as heparin, which may not accurately mimic the aggregation process in the human brain.^[5]^ These artificial systems fail to recapitulate the influence of the complex cellular environment, such as the role of co‐factors, chaperone proteins, and post‐translational modifications that modulate tau aggregation *in vivo*. Furthermore, although advanced techniques such as single‐molecule fluorescence imaging, cryo‐electron microscopy (cryo‐EM), and mass spectrometry (MS) are improving, they are still limited in their ability to probe the full spectrum of tau aggregates, particularly smaller, dynamic species and those in live systems where aggregation is transient and highly dynamic. As a result, our ability to fully elucidate the pathological significance of early‐stage tau aggregation remains constrained.

Addressing these methodological gaps is critical for advancing our understanding of tau aggregation and developing effective therapeutic strategies. Enhanced detection methods capable of accurately identifying small oligomeric tau species in living cells or human tissue are needed to better elucidate the complex dynamics of tau misfolding and its contribution to neurodegeneration.

## 2 General Overview of the Tau Protein

Tau is a highly diffusible, hydrophilic protein that belongs to the family of microtubule‐associated proteins (MAPs). It is predominantly expressed in neurons of the central nervous system (CNS), where it plays an important role in maintaining the structural integrity of axons by stabilising microtubules.^[34]^ Specifically, tau binds at the interface between α‐β‐tubulin heterodimers,^[35]^ thereby promoting microtubule assembly and stabilisation. This stabilising function is regulated by phosphorylation, which modulates the binding affinity of tau to microtubules.[^2^, ^3^]

**Scheme 2:**
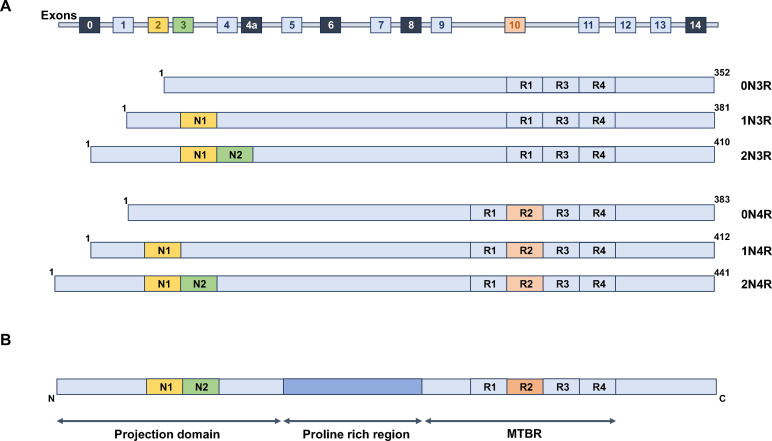
Alternative splicing of the MAPT gene generates six major tau isoforms in the adult human brain. (A) The MAPT gene undergoes alternative splicing of exons 2, 3, and 10, resulting in six tau isoforms with variations in N‐terminal inserts (0 N, 1 N, or 2 N) and microtubule‐binding repeats (3R or 4R). Exons 2 and 3 (yellow and green) encode the N‐terminal inserts, while exon 10 (orange) determines the presence of the second repeat (R2). (B) Tau's structure includes a projection domain (with N‐terminal inserts), a proline‐rich region, and the microtubule‐binding repeat region (MTBR) with either three (R1, R3, R4) or four (R1, R2, R3, R4) repeats.

Tau protein is encoded by the *MAPT* gene located on chromosome 17, which undergoes alternative splicing to produce six major isoforms in the adult human brain. These isoforms differ in the number of microtubule‐binding repeat domains (three or four repeats, 3R or 4R) and the number of N‐terminal inserts (zero, one, or two inserts, Scheme 2A).^[36]^ These isoforms are generated by alternative splicing of exons 2, 3, and 10. In healthy neurons, the balance between these isoforms is tightly regulated, with disturbances in the 3R:4R ratio being directly linked to pathological conditions.^[37]^ For instance, in certain tauopathies, an overrepresentation of one isoform over the other is observed and may contribute to disease pathogenesis. Mutations affecting exon 10 splicing have been implicated in familial tauopathies such as PSP, CBD, and frontotemporal dementia with parkinsonism linked to chromosome 17 (FTDP‐17).[^38^, ^39^] The tau protein comprises the N‐terminal projection domain, a proline‐rich region, and a microtubule‐binding repeat (MTBR) region (Scheme 2B).

Although tau's primary role is to stabilise microtubules, recent studies have revealed that tau may also perform other functions in neurons. Tau is involved in the regulation of axonal transport by regulating motor proteins such as kinesins and dyneins.^[40]^ Tau helps to ensure the efficient movement of vesicles, mitochondria, and other cellular components along microtubules. Abnormal tau modifications or mislocalisation can disrupt this transport system, potentially leading to neuronal dysfunction and degeneration.[^41^, ^42^] In addition to its structural role, tau has also been shown to have DNA‐ and RNA‐binding capabilities, which may protect nucleic acids from damage under conditions of cellular stress, such as oxidative stress.[^43^, ^44^, ^45^] These non‐structural functions of tau add another layer of complexity to its biology and highlight how dysregulation of tau in disease can affect multiple cellular processes.

### 2.1 Post‐Translational Modifications of Tau

Tau's function is strongly influenced by its post‐translational modifications (PTMs), which regulate its localisation, interaction with microtubules, and propensity to aggregate. Phosphorylation is the most extensively studied PTM of tau, with 85 phosphorylation sites identified (full‐length tau).^[46]^ In healthy neurons, tau is transiently phosphorylated, and this modification is required for certain physiological functions, such as facilitating microtubule dynamics during cell division.[^47^, ^48^] However, in pathological conditions, tau becomes hyperphosphorylated. While brain‐derived PHFs in healthy neurons may be phosphorylated at 2–3 moles of phosphate per mole of tau, AD‐brain‐derived PHFs can contain an average of 5–9 phosphate groups per molecule of tau.^[49]^ Phosphorylation at specific residues, such as Ser202,[^50^, ^51^] Thr231,^[52]^ and Ser396/404,[^53^, ^54^] are commonly linked to disease. However, different tauopathies have distinct phosphorylation profiles^[55]^ suggesting potentially different pathological mechanisms.

Tau hyperphosphorylation leads to a decrease in its affinity for microtubules and an increased tendency to self‐aggregate into toxic oligomers and fibrils.[^2^, ^3^, ^9^] Several kinases, including GSK‐3β^[56]^ and CDK5,^[57]^ are key regulators of tau phosphorylation. Both are dysregulated in AD and contribute to the abnormal phosphorylation of tau. In contrast, phosphatases such as PP2A are less active in AD,^[58]^ further promoting hyperphosphorylation. This imbalance between increased kinase and decreased phosphatase activity leads to hyperphosphorylation, which is central to the pathological aggregation of tau.

Other PTMs such as acetylation, ubiquitination, glycation, and nitration also play significant roles in modulating tau's behaviour. For example, tau acetylation inhibits its degradation by the proteasome, contributing to the accumulation of pathological tau species.^[59]^ Ubiquitination is involved in marking tau for degradation; however, in disease states, this system may be overwhelmed or dysfunctional, allowing misfolded tau to accumulate.[^60^, ^61^] Glycation and nitration, commonly associated with oxidative stress, further exacerbate tau misfolding and aggregation.[^62^, ^63^, ^64^, ^65^] The interplay between these various modifications underpins the multifactorial nature of tau‐related neurodegenerative diseases, making it a complex target for therapeutic intervention.

In healthy neurons, tau is predominantly localised in axons, where it performs its microtubule‐stabilising functions. However, in many neurodegenerative diseases, tau becomes mislocalised to the somatodendritic compartment of neurons.[^66^, ^67^] This mislocalisation is thought to contribute to tau pathology by promoting its aggregation. Tau mislocalisation also disrupts microtubule stability in axons, impairing axonal transport and synaptic function and leading to neurodegeneration.[^67^, ^68^] Additionally, mislocalised tau has been linked to the disruption of synaptic signalling. Studies have shown that tau can interact with postsynaptic proteins, such as the NMDA receptor, potentially altering synaptic plasticity.^[69]^ These effects may contribute to the cognitive deficits observed in tauopathies, particularly Alzheimer's disease, where tau pathology spreads from the entorhinal cortex to other brain regions involved in memory and learning.

### 2.2 Nomenclature and Definitions of Tau Aggregates

Tau aggregation involves a wide range of intermediates that precede the formation of mature fibrils. These intermediates, collectively referred to as diffusible or “soluble” tau aggregates, are increasingly recognised as key drivers of neurotoxicity in tauopathies. The term “diffusible” reflects their ability to remain dispersed in aqueous environments without sedimenting under mild centrifugation conditions, distinguishing them from larger, insoluble fibrils and neurofibrillary tangles. While the term “soluble aggregate” is often used, it is misleading since they do not dissolve in water but instead are aqueous extractable and diffusible. Various other terms have been used to describe these aggregates, including “low‐n oligomers”, “high‐n oligomers”, “protofibrils”, “short filaments”, and “protofilaments”. The lack of consensus in their nomenclature and classification can lead to confusion and hinder cross‐study comparisons.

Diffusible tau aggregates can generally be classified into three main categories based on their size, morphology, and degree of aggregation although these properties are more easily measured on *in vitro* aggregates than brain‐derived aggregates:

1. Low‐n Oligomers: These are small aggregates consisting of a few tau monomers (typically 2–5). They are often transient and may exist in a dynamic equilibrium with monomeric tau. Low‐n oligomers are characterised by a lack of stable β‐sheet structure and are less prone to self‐propagation. These aggregates are hypothesised to play a role in the early stages of tau aggregation. They can diffuse freely in the cellular environment.
2. High‐n Oligomers: These are larger, diffusible aggregates comprising dozens of monomers. High‐n oligomers are often stabilised by β‐sheet‐rich structures, making them more resistant to disaggregation. Despite their size, high‐n oligomers remain in aqueous solution under physiological conditions, likely due to the partial exposure of hydrophilic regions and incomplete β‐sheet formation. Their size and biochemical properties allow them to interact with cellular components, leading to toxicity. High‐n oligomers are commonly implicated in synaptic dysfunction and membrane disruption.
3. Protofibrils or Short Filaments: These intermediates are elongated aggregates with partial β‐sheet structure on the path to forming mature fibrils. Protofibrils are a single fibril protofilament and are structurally related to fibrils. They can seed the formation of fibrils and are considered a transitional species between diffusible aggregates and insoluble tau fibrils.

## 3 Protein Aggregation Processes

**Scheme 3:**
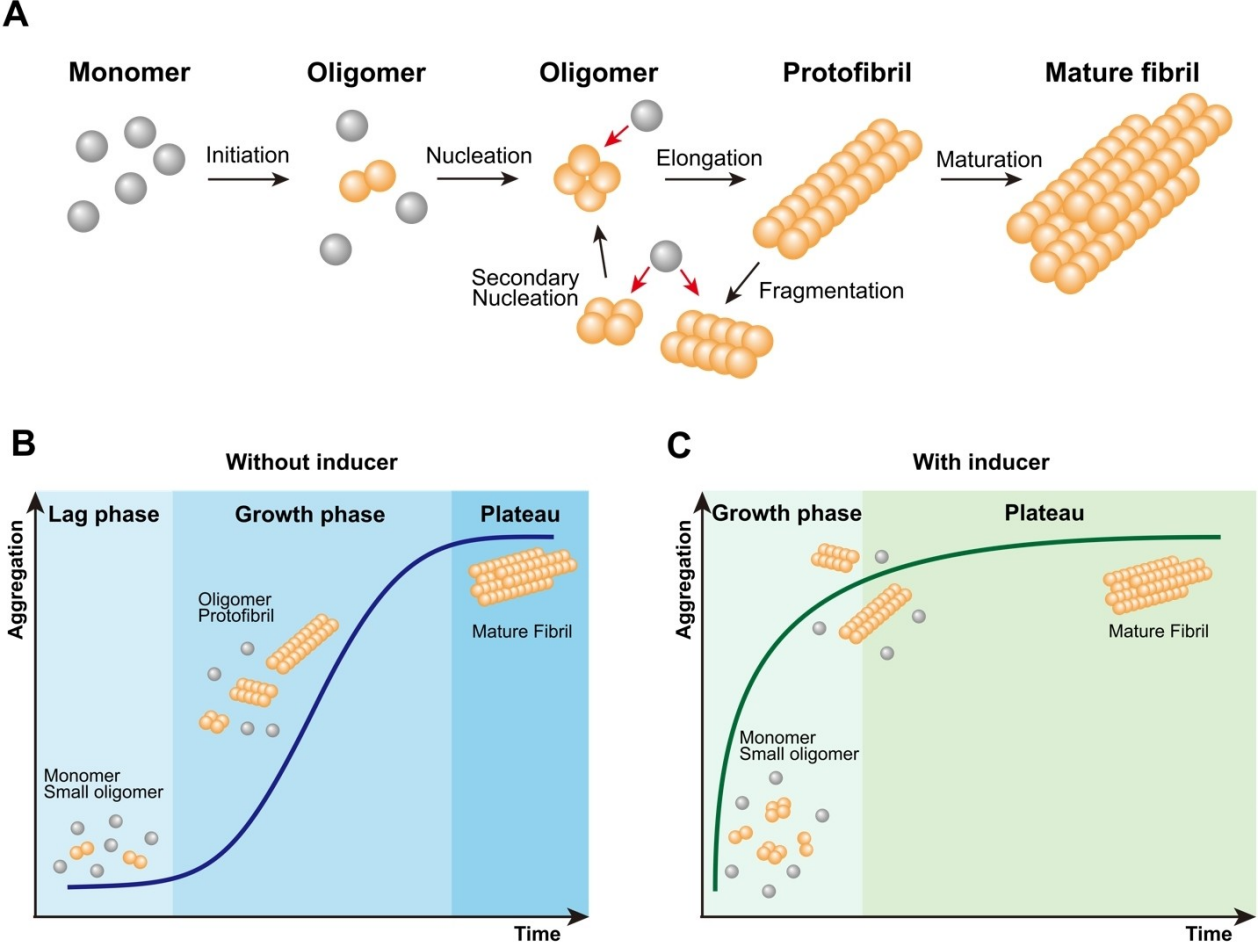
Process of aggregate formation. (A) Schematic representation of the tau aggregation process. Monomeric tau initially forms small oligomers, which elongate into protofibrils upon monomer addition. Secondary nucleation and fragmentation occur, amplifying the process by forming new oligomers from existing aggregates. The final step involves maturation, where the fibrils become insoluble and highly ordered. (B) Tau aggregation comprises three phase: the lag phase, the growth phase and a plateau phase. The initial formation of stable nuclei is the rate‐limiting step, determining the overall rate of aggregation. Once nucleation occurs, the process enters the growth phase, where elongation and secondary nucleation processes drive rapid fibril formation. Finally, the process reaches a plateau phase, where most tau is present as mature fibrils, and aggregation slows as available monomers are depleted. (C) The presence of aggregation inducers such as seeds, heparin, or *in vitro* hyperphosphorylation at certain sites^[71]^ accelerates the process by reducing or bypassing the lag phase. The inducer promotes immediate oligomer and protofibril formation, leading to faster growth of mature fibrils. This results in a shortened aggregation process with a rapid transition to the plateau phase.

Protein aggregation, including tau aggregation, follows a complex multi‐step process that is characterised by several distinct stages. Diffusible tau oligomers serve as precursors to fibrils, undergoing structural rearrangements to form β‐sheet‐rich protofibrils and, ultimately, insoluble fibrils. This general mechanism can be broadly divided into nucleation, elongation, and maturation phases, each involving different molecular events that drive the conversion of diffusible proteins into insoluble aggregates^[70]^ (Scheme 3A).

### 3.1 Nucleation

Nucleation is considered to be the rate‐limiting step in the aggregation process, involving the initial misfolding of protein monomers, which then interact to form small, unstable clusters known as nuclei or seeds. In the case of tau, under normal physiological conditions, the protein is highly diffusible and exhibits a natively unfolded structure. However, due to factors such as hyperphosphorylation, tau can undergo a conformational change that exposes its hydrophobic regions, allowing it to self‐associate. This phase is often slow because the formation of stable nuclei is thermodynamically unfavourable.[^72^, ^73^] However, once a critical nucleus is formed, the aggregation process becomes self‐propagating. Importantly, external factors such as aggregation inducers (e. g., heparin), oxidative stress, or seeding from preformed aggregates can accelerate nucleation and trigger the aggregation cascade.

### 3.2 Elongation

Once nuclei or seeds are formed, additional protein monomers rapidly bind to the growing ends of the seed, forming protofibrils or oligomers as intermediate species. Elongation is typically much faster than nucleation because the presence of stable nuclei lowers the energy barrier for aggregation. This process continues until protofibrils are formed, which are long, unbranched structures composed of aligned monomers in a β‐sheet‐rich conformation.

### 3.3 Secondary Nucleation and Fragmentation

Tau fibrils can amplify aggregation through two distinct secondary processes: surface‐catalysed secondary nucleation and fragmentation. In surface‐catalysed secondary nucleation, existing fibrils act as templates to nucleate the formation of new aggregates by recruiting additional monomers onto their surfaces.^[74]^ This process significantly accelerates fibril formation and contributes to the amplification of aggregation within affected regions.

Fragmentation, on the other hand, involves the physical breakage of fibrils into smaller fragments.^[75]^ These fragments serve as new seeds for aggregation, enhancing the overall fibril population and enabling the spread of tau pathology to distant regions of the brain. While both processes promote aggregation and pathology propagation, they are mechanistically distinct, with surface‐catalysed secondary nucleation focusing on monomer recruitment and fibril elongation, and fragmentation increasing the number of active fibril ends capable of seeding new aggregates.

### 3.4 Maturation

In the maturation phase, fibrils become more ordered and stable, forming insoluble aggregates. Tau, for instance, assembles into PHFs and SFs. These fibrillar structures eventually mature into the larger aggregates known as NFTs.

### 3.5 Seeding and Spreading

Tau is thought to be able to propagate its misfolded state in a prion‐like manner.[^76^, ^77^, ^78^] Aggregated forms of tau can act as seeds that are taken up by neighbouring cells and spread the aggregation process across different brain regions.

## 4 Studying Tau Aggregation in Disease: From *in vitro* to *in vivo*

Despite the critical importance of understanding the mechanisms of tau aggregation, these mechanisms are not very well understood yet. Studies of tau aggregation typically employ either *in vitro* or *in vivo* approaches. *In vitro* studies allow tau aggregation to be studied under relatively simple and controlled conditions, such as in test tubes and in cells, and can be used to understand aggregation kinetics and the impact of specific PTMs. However, these approaches are limited by the difficulty of inducing aggregation and the limitations of current models, which do not fully reflect the disease. In contrast, *in vivo* studies typically focus on tau aggregation in mice or post‐mortem brain tissue, which are closer to tauopathy development; nevertheless, *in vivo* studies are often hindered by the lack of detection methods with satisfactory sensitivity and specificity in complex samples, as well as the inability to probe tau aggregation in real time.

To better understand disease pathogenesis, many studies focus on the nucleation steps of tau aggregation. Yet, due to the limitations of current methods in studying these processes within biological systems, most studies are limited to replicating them *in vitro*. However, the aggregation of wild‐type (wt) full‐length tau under physiological conditions presents significant challenges. The high solubility of tau and its resistance to spontaneous fibril formation under physiological conditions make it difficult to replicate the aggregation process observed *in vivo*.^[79]^ Consequently, many studies use cofactors, mechanical agitation, in vitro hyperphosphorylation, seeds, or truncated or mutant forms of tau to initiate aggregation (Scheme 3B, C).

Several strategies have been developed to promote tau aggregation *in vitro*. Many *in vitro* studies of tau aggregation often rely on the use of various cofactors, such as anionic substances like heparin,^[80]^ polyglutamic acids,^[81]^ nucleic acids,^[82]^ and negatively charged phospholipids,^[83]^ to trigger heterogeneous primary nucleation.^[11]^ Other methods, such as vigorous shaking,^[84]^ have also been employed to accelerate both primary and secondary aggregation processes. However, heparin and similar cofactors may not accurately mimic the physiological processes driving tau aggregation in the brain, as comparative studies have found differences in the conformations, properties, and activities of recombinant tau aggregates compared to AD‐derived aggregates.[^5^, ^85^] To address this limitation, more physiologically relevant cofactors, such as RNA^[86]^ and arachidonic acid,[^87^, ^88^] have been explored as triggers for tau aggregation *in vitro*. These approaches aim to provide better models of tau self‐assembly that are closer to the conditions found *in vivo*. Nonetheless, these artificial and often harsh induction systems may not adequately represent tau self‐assembly *in vivo*, which might be driven by aberrant phosphorylation and other PTMs.

To avoid the addition of cofactors, highly truncated forms of tau are frequently used in studies to overcome the resistance of full‐length tau to aggregation. For example, the tau amino acids (aa) 304–380 fragment has been shown to spontaneously assemble into fibrils under quiescent conditions in pure buffer systems.^[11]^ This study demonstrates that the aggregation mechanism of this tau fragment is primarily governed by secondary nucleation processes at the fibril surfaces, which serve as the dominant source of new oligomers. Similarly, the aa 297–391 fragment has been shown to spontaneously form filamentous aggregates whose structure resembles PHFs in AD patients.^[89]^ Although these truncated tau fragments aggregate without the need for cofactors or harsh conditions, they still differ from the tau aggregates observed in biological samples in morphology, kinetics of formation, and, potentially, functions.

Alternatively, the spontaneous self‐assembly into small aggregates of full‐length tau at physiological concentrations and without any external inducer has been achieved using hyperphosphorylated tau.[^9^, ^71^, ^90^] This hyperphosphorylation can be induced *in vitro* using specific kinases, such as PKA or GSK‐3β, that mimic disease‐relevant PTMs. These aggregates have been shown to elicit toll‐like receptor 4 (TLR4)‐dependent inflammatory responses, indicating that they may have a distinct biological activity *in vivo*.^[9]^ Notably, the self‐assembly of these *in vitro* hyperphosphorylated aggregates occurred without a detectable lag phase. While most studies indicate that hyperphosphorylation generally promotes tau aggregation, it is important to note that specific phosphorylation patterns can inhibit aggregation[^91^, ^92^] highlighting the complex interplay between phosphorylation and tau aggregation dynamics. This complexity underscores the need for caution when interpreting the aggregation behaviour of hyperphosphorylated tau, as the specific pattern of phosphorylation may significantly influence the process.

Significant differences in the self‐assembly and behaviour of *in vitro* hyperphosphorylated tau aggregates have been observed compared to heparin‐induced aggregates, with the latter being more efficient at seeding further aggregation.^[71]^ However, concerns about using *in vitro* hyperphosphorylated tau include the lack of specificity of enzymatic phosphorylation, where kinases often target multiple tau residues, leading to a mixture of differentially phosphorylated proteins. This variability raises questions about the reproducibility and physiological relevance of the resulting aggregates. It also remains unclear how closely these *in vitro* aggregates resemble those found *in vivo*, complicating efforts to accurately model tau aggregation.

*In vivo* studies using animal models have advanced our understanding of tau aggregation. Models such as mice expressing aggregation‐prone mutant human tau have successfully replicated several key aspects of tau pathology, including NFTs,^[93]^ tau oligomers,^[94]^ and tau‐positive inclusions.^[95]^ These models also exhibit behavioural deficits such as memory impairment and motor dysfunction, which further resemble the symptoms of human tauopathies.[^96^, ^97^, ^98^] One of the significant findings from these models is the correlation between tau propagation and synaptic and neuronal loss, suggesting that tau aggregates actively contribute to neurotoxicity rather than merely accumulating passively. Advanced imaging techniques, such as multi‐photon microscopy, have recently enabled real‐time visualisation of tau aggregation in live animals.^[99]^ For example, luminescent conjugated oligothiophenes have been used to identify and track the formation and progression of tau fibrils and Aβ aggregates, revealing dynamic interactions between these pathological species, their spatial distribution, and their impact on neuronal function over time.^[99]^

Despite these advancements, these models also face significant limitations in accurately replicating human tauopathies. Firstly, murine tau does not naturally form fibrils with age, and only a few mouse models show accumulation of endogenous murine Aβ.^[100]^ Even the most aggressive models, such as the 5xFAD mouse, which rapidly accumulates Aβ and develops early plaques, do not exhibit secondary tau tangle formation or significant neuronal loss as seen in human AD.^[101]^ Consequently, most commonly used transgenic mouse models rely on the expression of mutant human tau protein, such as P301L and P301S,^[102]^ which are associated to rare, early‐onset forms of tauopathies. These models tend to exhibit much more aggressive disease progression than seen in typical human tauopathies. Furthermore, these models usually express only one tau isoform, typically 4R tau, ignoring the critical role of isoform diversity and alternative splicing in disease processes.[^103^, ^104^] Additionally, late‐stage modifications such as tau ubiquitination and acetylation are often inadequately represented.^[105]^ The resulting pathology, while informative, does not fully replicate the tau modifications observed in human diseases. This limits the utility of these models for studying the full progression of tau pathology or for testing drug candidates targeting advanced disease stages.

To address these limitations, alternative approaches are being explored. Seeding‐based mouse models, in which pathological tau from post‐mortem human brains is injected into non‐transgenic mice, have shown promise in more accurately replicating the patterns of tau pathology observed in human diseases such as AD, CBD, and PSP.[^106^, ^107^] Additionally, more sophisticated *in vitro* models, such as organoids and induced neurons (iNeurons),[^108^, ^109^] are being developed. These models allow the interaction between human microglia, astrocytes, and neurons to be studied, providing a more comprehensive understanding of tau pathology.

To summarise, *in vitro* studies of tau aggregation often rely on truncated tau and non‐physiological conditions and are thus limited in their ability to accurately model tau aggregation. While *in vivo* models offer a more realistic representation of tau aggregation by incorporating PTMs and other cellular regulatory mechanisms, they also come with their own set of challenges. The discrepancies between *in vitro* and *in vivo* models, as well as the gap between these models and human tauopathies, highlight a critical challenge for drug discovery, as compounds that inhibit tau aggregation *in vitro* may not have the same efficacy or mechanism of action in a living organism.

## 5 Methods to Detect Tau Aggregation

While current *in vitro* and *in vivo* models have provided valuable insights into tau aggregation and its role in neurodegenerative disease, they often fall short in capturing the early and subtle stages of aggregation, which are critical for understanding disease initiation and progression. Moreover, the heterogeneity and low concentration of tau aggregates in biological samples present significant challenges to traditional detection methods.[^32^, ^88^, ^110^, ^111^] To overcome these barriers and advance our understanding of tauopathies, it is essential to develop and refine techniques that can sensitively and specifically detect and characterise tau aggregates. These techniques are expected to probe the concentration, size, structure, and biological functions of heterogeneous tau aggregate species in a complex biological sample.

One of the major challenges in tau pathology is the early detection of small tau aggregates, particularly oligomers, which have been shown to be neurotoxic.^[12]^ Moreover, detection of these early‐stage aggregates offers the potential for earlier diagnosis, allowing intervention at a stage when therapeutic strategies may be more effective before significant neuronal loss occurs. However, due to their small size and low abundance, most methods struggle to detect these small aggregates and capture their full heterogeneity. Thus, there is a need for more sensitive, specific detection methods that can identify tau species in their earlier, most toxic forms.

The following sections review the challenges in detecting and characterising tau aggregates and the advanced methods being developed to address these challenges, offering new opportunities to study tau aggregation with greater accuracy and relevance to human disease (Table 1).

**Table 1:**
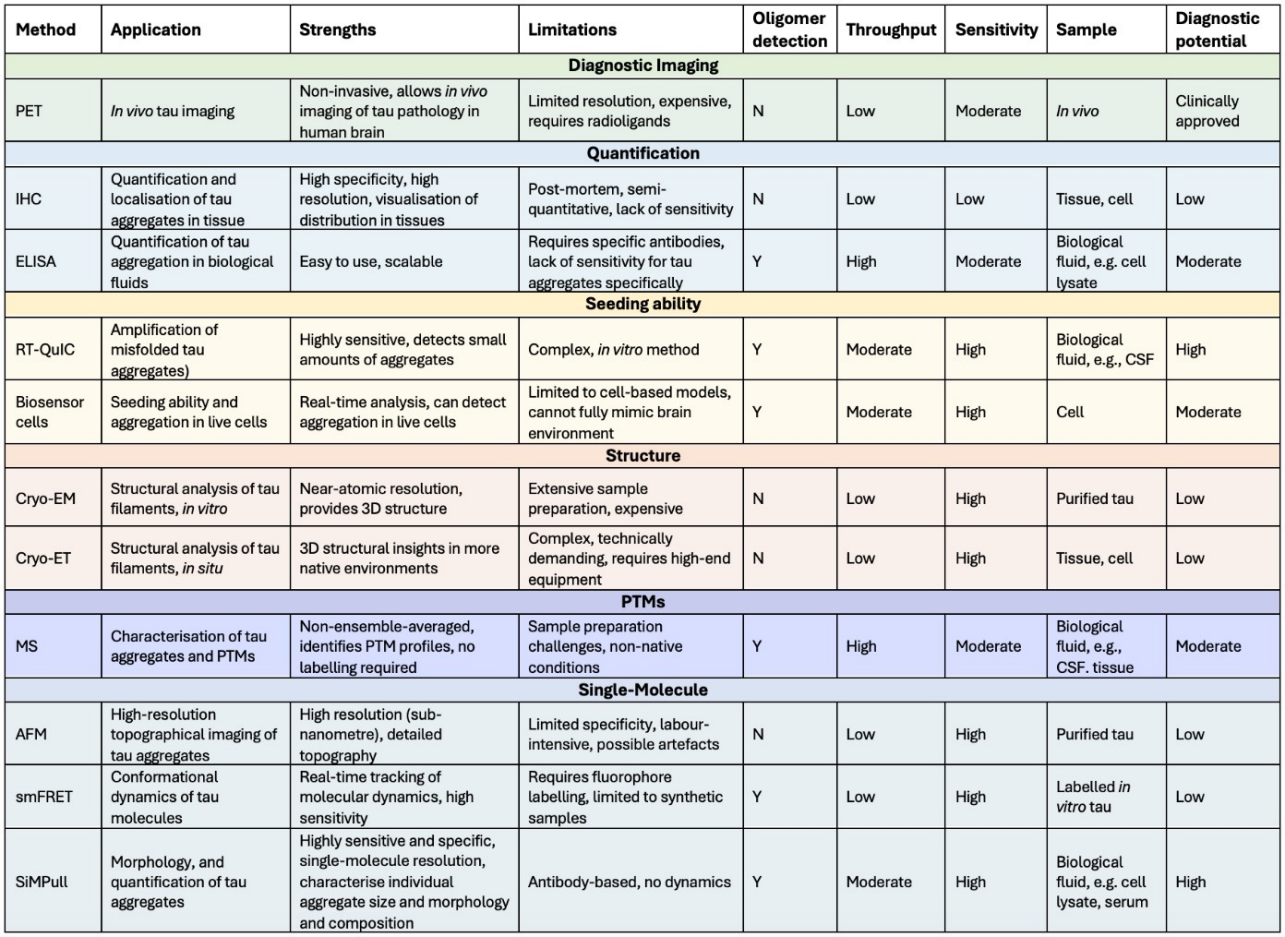
Overview of commonly used methods for detecting tau aggregation. PET, Positron emission tomography; IHC, Immunohistochemistry; ELISA, Enzyme‐linked immunosorbent assay; RT‐QuIC, Real‐time quaking‐induced conversion; CSF, Cerebrospinal fluid; Cryo‐EM, Cryo‐electron microscopy; Cryo‐ET, Cryo‐electron tomography; PTM, Post‐translational modification; MS, Mass spectrometry; AFM, Atomic force microscopy; smFRET, Single‐molecule Förster resonance energy transfer; SiMPull, Single‐molecule pull‐down.

### 5.1 Positron Emission Tomography (PET)

Positron emission tomography (PET) is a favoured methodology for imaging global tau aggregate burden *in vivo*, using a ‘tracer’ dye to report on the density and distribution of tau protein deposits in the brains of people living with AD.^[112]^ PET scans are typically integrated with computed tomography (CT) or magnetic resonance imaging (MRI) scanners. PET tracers are radiolabelled compounds, often small molecules or peptides, designed to selectively bind to the structural features of tau aggregates. These tracers typically include a positron‐emitting isotope, such as fluorine‐18, and bind to beta‐sheet structures within tau aggregates, enabling their visualisation during the scan.^[113]^ However, current PET tracers only bind end‐stage tau aggregates formed in advanced disease and have been shown to have a weaker affinity for tau aggregates in non‐AD tauopathies.^[114]^ Additionally, PET tracers do not provide sufficient sensitivity to detect the early onset of disease when tau aggregates are small and low in concentration, and they cannot resolve these aggregates at the cellular level due to low spatial resolution. PET imaging is very expensive (several thousand USD per scan), making it unsuitable as a screening tool for early diagnosis.

### 5.2 Immunostaining

Immunostaining is a widely used method for visualising tau, using antibodies that specifically bind to tau protein to detect its presence in the sample. This technique enables the localisation and quantification of tau accumulations, making it useful for studying disease pathology in post‐mortem samples. However, immunostaining faces several limitations, particularly in detecting early‐stage tau aggregates. Immunostaining often lacks the resolution needed to detect the small tau aggregates, which are typically smaller than the diffraction limit of light (~250 nm). There is also generally a high autofluorescence background in post‐mortem tissue making it difficult to detect weak fluorescence signals.[^115^, ^116^] Additionally, most antibodies used in immunostaining are designed to bind to larger, mature tau filaments, resulting in reduced sensitivity to smaller tau species. Oligomer‐specific antibodies, such as TOMA^[117]^ or TOC1,^[118]^ also present significant challenges as they are often developed to recognise specific conformations of tau, limiting their effectiveness in detecting the heterogeneous structures of smaller aggregates. It is worth noting that the conformational diversity among aggregates can make it difficult for antibodies to target early‐stage aggregates; also, conformation‐specific antibodies may show cross‐reactivity with other protein aggregates or monomers,[^119^, ^120^] reducing their overall specificity for tau. Furthermore, antibodies designed to detect tau oligomers are often not well characterised nor validated for consistent performance.^[121]^ This inconsistency in the detection and quantification of toxic tau species hinders the accurate assessment of early tau pathology and disease progression.

### 5.3 Enzyme‐Linked Immunosorbent Assay (ELISA)

Enzyme‐linked immunosorbent assay (ELISA) is another widely used technique for detecting and quantifying tau protein in biological samples.^[122]^ ELISA is a type of immunoassay that utilises antibodies to specifically capture and detect a protein of interest (POI), thereby quantifying its concentration in a liquid biological sample. ELISA is suitable for detecting POIs at low concentrations (pM~nM) by using detector antibodies conjugated with enzymes, which catalyses the conversion of fluorogenic substrates, to amplify the signals from the captured POI.^[123]^ ELISA can provide quantitative measurements of tau levels in a wide range of samples, including tissue, cell extracts and clinical biofluids.[^122^, ^124^, ^125^] While standard ELISA is effective in detecting total tau or phosphorylated tau,^[126]^ it faces significant limitations when it comes to detecting tau aggregates. While aggregate‐specific ELISA assays are available,^[127]^ their sensitivity is often insufficient for detecting low concentrations of tau aggregates, and they are therefore limited to a small range of sample types. This limits their utility for early diagnosis and for studying the initial stages of tau aggregation in disease development.

A more recent advancement is the use of single‐molecule array (Simoa), which is often described as a digital ELISA. Simoa achieves higher sensitivity by isolating and detecting individual enzyme‐labelled immunocomplexes in femtoliter‐sized wells, allowing for quantification at much lower concentrations than conventional ELISA (typically 1000‐fold).^[128]^ This increased sensitivity has made Simoa particularly valuable for detecting tau and phosphorylated tau in serum and plasma samples, enabling the study of tau biomarkers in minimally invasive biofluids and advancing the early diagnosis of tauopathies.[^129^, ^130^, ^131^] Additionally, Simoa assays have recently been developed for the detection of soluble tau aggregates, providing a powerful tool for studying tau aggregates in human brain homogenate[^132^, ^133^] and blood.^[133]^

### 5.4 RT QuIC

Several techniques have also been developed to detect and study tau aggregates in a range of biofluids. Highly sensitive tau seeding assays (Real‐Time Quaking‐Induced Conversion; RT QuIC), originally developed for prion diseases, can detect the relative aggregation potential of tau in post‐mortem brain samples and cerebrospinal fluid (CSF)[^134^, ^135^] by amplifying low levels of seeds. This method uses multiple rounds of fibril growth and fibril fragmentation to produce detectable signals.

### 5.5 Biosensor Cells

Biosensor cells are capable of detecting tau aggregation by utilising genetically engineered cell lines that express tau fused to fluorescent reporters. These cells are designed to detect and report on the formation and propagation of tau aggregates in real time, offering insights into tau seeding and the spread of aggregation within a cellular environment^.[136]^ One widely used approach involves fluorescence resonance energy transfer (FRET) or bioluminescence resonance energy transfer (BRET), where the close proximity of aggregated tau proteins induces a measurable energy transfer between the fluorescently tagged tau molecules. This change in fluorescence intensity can be quantified, allowing for the detection of tau aggregation dynamics.[^136^, ^137^, ^138^]

Many biosensor cell models rely on the expression of truncated or aggregation‐prone mutant forms of tau, which increase the efficiency of aggregation and seeding. For example, tau fragments encompassing the repeat domain of tau (RD tau, residues 244–372),^[136]^ are commonly used in biosensor systems due to their enhanced aggregation, as they lack the N‐terminal and proline‐rich regions of full‐length tau, which normally maintain solubility.^[139]^ The P301S mutation is one of the most commonly used in these assays, applied to both truncated^[136]^ and full‐length^[140]^ tau. However, it remains unclear whether the conformations resulting from seeding onto P301S tau in cell culture accurately replicate those found in human disease.^[141]^ Assays using wt full‐length tau have also been developed, providing a more physiologically relevant system for studying tau aggregation and its propagation in sporadic disease.^[142]^

Tau biosensor cells have been utilised to detect and quantify bioactive tau from diverse Alzheimer′s disease samples, including purified brain extracts and CSF.[^143^, ^144^, ^145^] They enable live‐cell monitoring of tau aggregation, seeding, and propagation in response to external tau seeds or other aggregation inducers. This real‐time monitoring offers an opportunity to study the cell‐to‐cell transmission of tau aggregates.[^137^, ^146^] Furthermore, studies using biosensor cells have shown that high molecular weight tau species (>2,000 kDa) have the strongest seeding activity.^[147]^ While biosensor cells are highly sensitive to tau seeding activity, they face limitations in mimicking the full complexity of human brain tissue, where tau aggregates interact with various cell types and environments. Additionally, these models may not fully replicate the specific pathological conditions of different tauopathies. Despite these limitations, biosensor cells are a powerful tool for screening compounds that target tau aggregation and for studying the mechanistic aspects of tau spreading in disease models.

### 5.6 Cryo‐Electron Microscopy (cryo‐EM)

Cryo‐electron microscopy (cryo‐EM) has recently emerged as a powerful imaging technique, elucidating the structure of tau aggregates at near‐atomic resolution.^[4]^ By flash‐freezing tau‐containing samples, cryo‐EM preserves their native structure and allows for high‐resolution imaging with an electron microscope.^[148]^ Cryo‐EM has provided critical insights into the morphology and assembly of tau filaments, revealing distinct structural conformations associated with different tauopathies. For example, cryo‐EM studies have shown that tau filaments in Alzheimer's disease exhibit a different conformation than those found in Pick's disease or chronic traumatic encephalopathy (CTE).[^22^, ^149^] These structural differences are thought to contribute to the distinct clinical and pathological features of each disease.^[20]^ By identifying these unique structural ‘signatures’, cryo‐EM has the potential to improve disease classification and deepen the understanding of how tau aggregation contributes to disease progression.

Although cryo‐EM provides detailed structural information, the need for highly purified samples^[148]^ currently limits its application in clinical diagnostics. Another major limitation of cryo‐EM is its inability to reconstruct smaller, non‐fibrillar tau species such as oligomers due to their high heterogeneity (rather than their size). While cryo‐EM excels at resolving the structure of highly ordered fibrillar forms of tau, it is less effective at identifying early‐stage aggregates that are less well‐structured.[^150^, ^151^] Moreover, although cryo‐EM offers exceptional resolution, it primarily provides static images of the tau aggregates, which may not fully capture the dynamic process of tau aggregation as it occurs in living cells or over time during disease progression. As a result, cryo‐EM is currently more suited to advancing fundamental research into tau aggregation and its molecular mechanisms rather than serving as a clinical tool for early diagnosis or monitoring of disease progression. Nonetheless, the insights gained from cryo‐EM are invaluable for informing the development of new therapeutic strategies aimed at targeting specific tau structures and preventing the toxic aggregation process.

### 5.7 Cryo‐Electron Tomography (Cryo‐ET)

Cryo‐electron tomography (cryo‐ET) is an advanced imaging technique that enables the structure determination of macromolecules in their native cellular context at a near‐atomic resolution.^[152]^ A recent study leveraged cryo‐ET to reconstruct individual tau filaments in post‐mortem AD brain tissue, specifically within neurons of the mid‐temporal gyrus, indicating subcellular heterogeneities in their structure and distribution.^[153]^ Additionally, subtomogram averaging can be applied in cryo‐ET to improve the resolution of specific repetitive features, such as tau filaments, allowing more detailed structural analysis. In the same study, the *in‐situ* structure of tau filaments within post‐mortem human brain tissue has been resolved with 8.7–31.8 Å resolution, showing spatially organised fibril heterogeneity.^[153]^ These insights into the spatial distribution and structural context of tau aggregates offer a clearer understanding of how tau filaments form and accumulate in different cellular compartments.

Beyond neurons, cryo‐ET has also been used to study tau filaments in extracellular vesicles (EVs).^[154]^ A recent study combined cryo‐ET, single‐particle cryo‐electron microscopy, and quantitative MS to investigate tau filaments within brain EVs from individuals with AD.^[154]^ This research revealed that the tau filaments, composed primarily of truncated tau, were enclosed within EVs enriched in endo‐lysosomal proteins. The filaments were observed tethered to the EV limiting membrane via specific molecular interactions, suggesting a selective packaging mechanism. These findings highlight the role of EVs in the secretion and propagation of tau, offering potential therapeutic and biomarker targets.

Despite its transformative impact, cryo‐ET faces several challenges that limit its widespread application. The technique requires specialised equipment and expertise, and its ability to detect small, early‐stage aggregates such as tau oligomers is still limited due to its difficulty in capturing less‐ordered structures compared to fibrils.^[151]^ Additionally, sample preparation for cryo‐ET is labour‐intensive, requiring vitrification and precise sectioning of cells or tissues, which makes it challenging to scale up for routine use. Despite these limitations, cryo‐ET has significantly advanced our understanding of tau aggregation by providing a detailed view of tau fibrils in their cellular context.

### 5.8 Mass Spectrometry (MS)

Mass spectrometry (MS) has emerged as a valuable tool in the characterisation of tau aggregates and their post‐translational modifications (PTMs). Unlike many traditional techniques, MS does not require dye labelling or immobilisation strategies,^[155]^ offering a non‐ensemble‐averaged approach to studying protein aggregation.^[153]^ When combined with complementary methods such as infrared spectroscopy, ion mobility, or chemical cross‐linking, MS can provide detailed structural insights into tau oligomers and fibrils.[^156^, ^157^, ^158^] Recent applications of MS have focused on identifying phosphorylation sites in tau from biological samples such as CSF, brain tissue, and blood plasma,^[159]^ revealing distinct PTM profiles associated with each.^[160]^ These findings are critical for understanding the molecular mechanisms driving disease progression and for identifying potential biomarkers. Recent advancements, such as charge detection mass spectrometry (CDMS), have extended the utility of MS to characterise larger aggregates of tau protein at the single‐molecule level.^[161]^ Additionally, mass photometry, an emerging technique, enables the monitoring of tau oligomerisation over time under native conditions.^[162]^ Although its mass resolution is lower than that of conventional MS, its ability to preserve native‐like environments provides valuable insights into aggregation kinetics.

Together, these approaches highlight the versatility of MS in addressing key challenges in tau research, from detecting low‐abundance species to elucidating structural details of pathological aggregates. While limitations remain, particularly in the preparation of physiologically relevant samples, ongoing developments are likely to enhance their application in understanding tau aggregation and its role in disease.

## 6 Single‐Molecule Methods to Characterise Disease‐Associated Aggregates

**Scheme 4:**
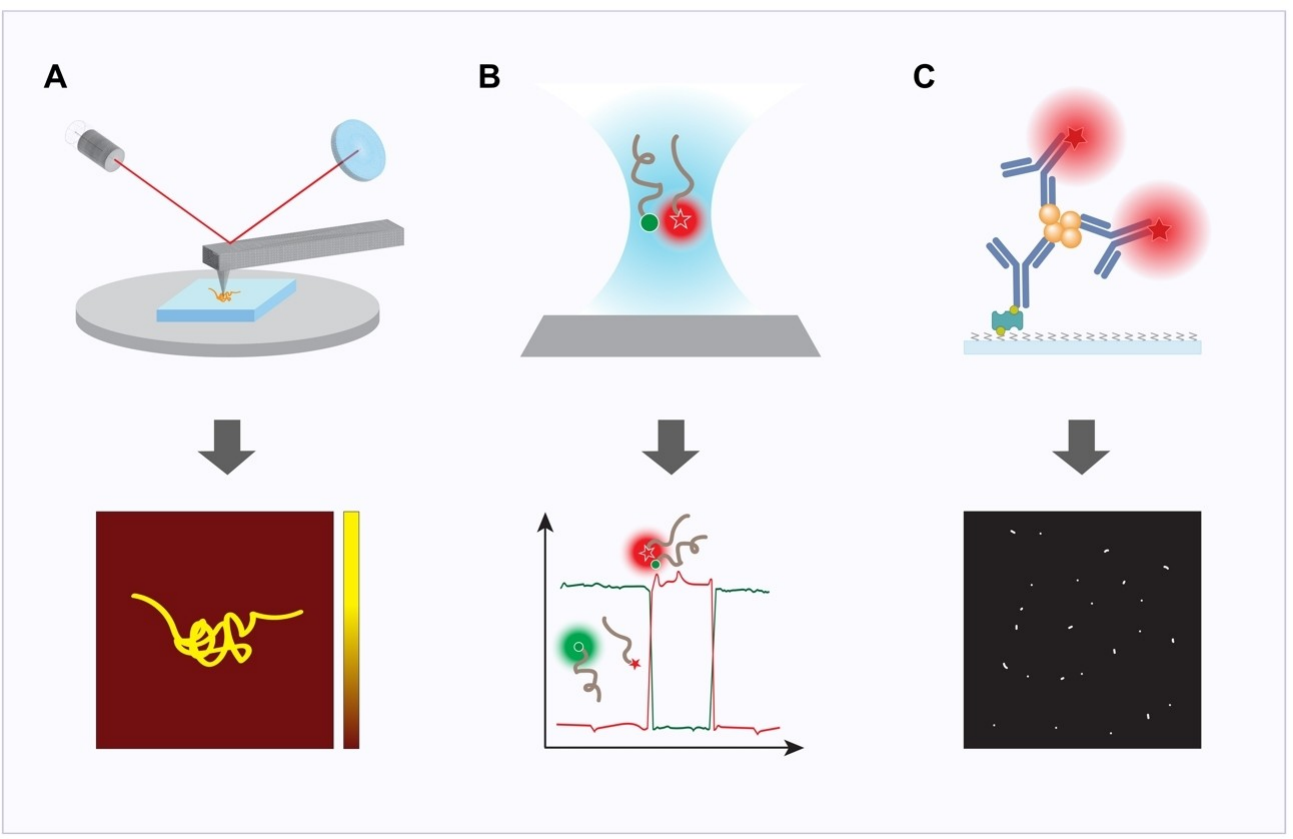
Overview of single‐molecule techniques used to study tau aggregation. (A) Atomic force microscopy (AFM): A cantilever scans the sample surface to generate high‐resolution topographical images of tau fibrils and aggregates. The resulting data (bottom) provide detailed structural information, allowing visualisation of individual tau filaments. (B) Single‐molecule Förster resonance energy transfer (smFRET): This technique uses energy transfer between donor (green) and acceptor (red) fluorophores to monitor real‐time conformational changes in tau molecules or aggregates. The resulting data (bottom) shows fluorescence intensity changes, enabling the study of tau folding and aggregation kinetics at the single‐molecule level. (C) SiMPull (Single‐molecule pull‐down): Tau aggregates are captured from complex biological samples using antibodies attached to a passivated surface. Fluorescently labelled tau aggregates are detected individually (bottom), allowing quantification and analysis of tau isoforms, aggregation state, and interactions.

Tau aggregates are highly heterogeneous in terms of size, shape, and phosphorylation state, which makes studying and quantifying them very challenging. The techniques introduced above are mostly bulk methods, i. e., they can only characterise the entire aggregate population and provide only an average thereof. As a result, these techniques cannot specifically identify and characterise the pathological tau aggregates, which represent only a small subset of the total aggregate population. In recent years, single‐molecule methods have emerged as critical tools in studying tau aggregation (Scheme 4). These methods allow for the analysis of individual aggregates, enabling the distinction between different tau aggregate subpopulations and the identification of pathological ones. Furthermore, some of these single‐molecule methods can provide statistical information about the aggregate population, such as size distribution, to facilitate clinical applications. With these methods, it is also possible to gain insight into the potential causality of specific characteristics resulting in tau pathology and the underlying mechanisms of disease.

### 6.1 Atomic Force Microscopy

Atomic force microscopy (AFM) is a powerful technique that has been widely applied in *in vitro* tau aggregation studies due to its ability to provide high‐resolution, three‐dimensional images of surfaces at the nanometre scale (Scheme 4A).^[163]^ AFM works by scanning a sharp probe, known as a cantilever, across the surface of a sample. The cantilever moves up and down in response to interactions between the probe and the sample, and these movements are recorded to produce detailed topographical maps of the surface. One of the key advantages of AFM is its sub‐nanometre resolution, which allows individual protein aggregates to be visualised. AFM has also proven valuable in biophysics and biomolecular research, with studies highlighting its potential for investigating protein interactions and aggregation dynamics.^[164]^ Additionally, AFM allows imaging under near‐physiological conditions, such as in buffer solutions, and even on live cells, providing more biologically relevant insights compared to methods requiring harsher sample preparations.[^165^, ^166^] While AFM has provided critical insights into the physical properties of tau aggregates and their growth over time,^[163]^ it is burdened by several limitations, including the lack of specificity. While it is possible to detect protein aggregates, it is not possible, for example, to distinguish between tau and amyloid beta, resulting in ambiguous results when analysing complex biological samples where multiple proteins are present. This limitation could potentially be addressed using antibody‐functionalised AFM tips, which allow for molecular recognition of specific proteins.^[167]^ However, the approach relies heavily on the specificity and quality of the antibodies used. Additionally, when applied to biological samples, the need to adsorb the sample onto grids complicates the process and often introduces artefacts.^[168]^ Moreover, AFM processes tend to be labour‐intensive, which can be a barrier to routine application.

### 6.2 Single‐Molecule Förster Resonance Energy Transfer

Single‐molecule fluorescence microscopy techniques, such as Single‐Molecule Förster Resonance Energy Transfer (smFRET), observe the fluorescence emitted by individual tau molecules or their aggregates (Scheme 4B).[^169^, ^170^] The principle behind smFRET relies on the energy transfer between two fluorophores (a donor and an acceptor) when they are in close proximity, typically within 1–10 nanometres. By placing the donor and acceptor within the same molecule, the distance between these fluorophores changes upon conformational changes, enabling the observation of real‐time molecular behaviour to track the dynamics, interactions, and conformational changes of individual tau species.^[171]^ For instance, smFRET has been used to study the heparin‐induced aggregation of the repeat region of tau, revealing several distinct subpopulations of tau oligomers with varying stabilities during the aggregation process.^[172]^ Some oligomers were found to be transient and kinetically unstable, with only a small fraction progressing towards fibril formation, while others, more stable but off‐pathway, could contribute to cellular toxicity by overwhelming protein quality control systems. However, current techniques are typically applied only to synthetic tau samples and do not rely on the specific capture of tau aggregates.[^173^, ^174^] Thus, their ability to handle biologically complex samples remain highly limited, restricting their applicability in studying disease progression in real‐world biological systems. Thus, there is a need for assays that can detect small tau aggregates with very high sensitivity and specificity in a range of biological samples on a single‐molecule level.

### 6.3 Single‐Molecule Pull‐Down

**Scheme 5:**
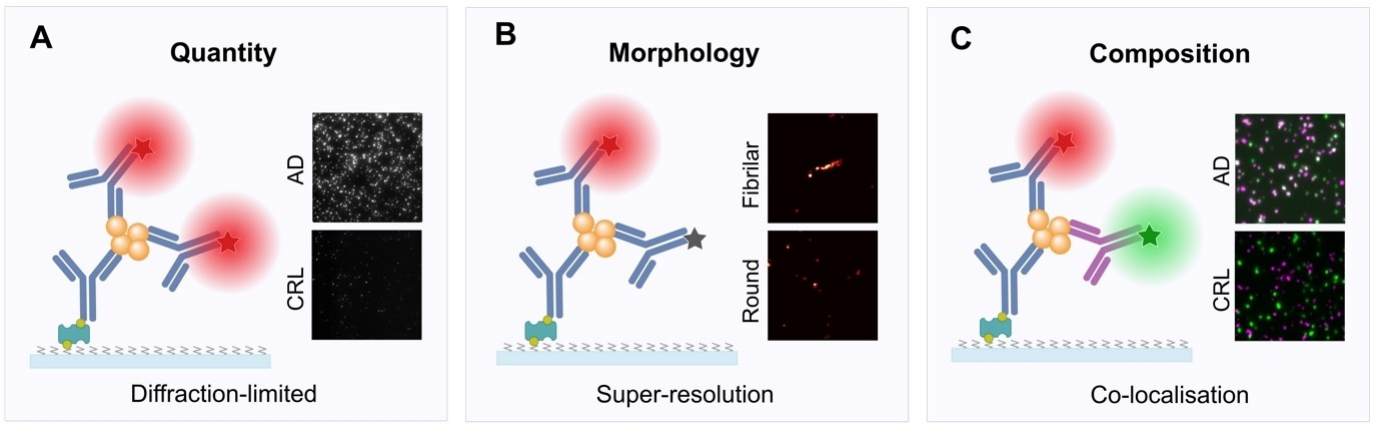
Detection and characterisation of tau aggregates using single‐molecule pull‐down (SiMPull). SiMPull can be adapted for the detailed analysis of tau aggregate quantity, morphology and composition. (A) The number of tau aggregates is determined using diffraction‐limited fluorescence microscopy. Representative images show a higher density of tau aggregates in post‐mortem brain homogenate from Alzheimer's disease samples compared to control. (B) Super‐resolution imaging enabled the detailed analysis of the morphology of tau aggregates, identifying fibrillar and round tau aggregates. (C) Co‐localisation of different post‐translational modifications of tau to identify differences in tau composition between disease and control samples.

Single‐molecule pull‐down (SiMPull) is an emerging technique that offers high sensitivity and specificity for studying individual protein aggregates, including tau (Scheme 4C).[^32^, ^175^] By using an antibody‐based capture method, SiMPull isolates specific tau species from complex biological samples onto a surface, enabling the direct visualisation of individual molecules or aggregates with pM to nM sensitivities.[^32^, ^176^] Specifically, SiMPull uses a functionalised glass coverslip to immobilise capture antibodies. The biological sample is loaded onto the coverslip, allowing tau aggregates to be captured while non‐binding proteins in the sample are then washed away. The captured tau aggregates are labelled with fluorescently labelled imaging antibodies. After the removal of excess imaging antibodies, the coverslip is imaged on a fluorescence microscope to characterise the number and morphology of tau aggregates. It should be noted that these tau aggregates can be visualised using conventional diffraction‐limited fluorescence imaging or super‐resolution imaging techniques, particularly single‐molecule localisation microscopy (SMLM) techniques. Diffraction‐limited imaging can provide information on the number and fluorescence intensity of these aggregates, but it cannot resolve small aggregates with sizes below the diffraction limit of light (Scheme 5A). SMLM techniques, such as (direct) stochastic optical reconstruction microscopy ((d)STORM) and DNA point accumulation in nanoscale topology (DNA‐PAINT), can further push the spatial resolution to ~20 nm, enabling the morphological characterisation of these small tau aggregates (Scheme 5B). SiMPull combines the concepts of traditional immunoprecipitation with single‐molecule fluorescence imaging, allowing for the analysis of specific protein interactions and conformational states at the single‐molecule level.

One of the key advantages of SiMPull is its ability to selectively capture tau aggregates while excluding other proteins, thereby overcoming one of the major limitations of other single‐molecule techniques, such as AFM, which lack protein specificity. This specificity allows tau aggregates to be distinguished from other proteins such as amyloid‐beta, which is critical in the context of tauopathies and mixed‐pathology diseases like Alzheimer's disease.

An adaptation of SiMPull, known as surface‐based fluorescence intensity distribution analysis (sFIDA), enhances the quantitative analysis of tau aggregates.^[177]^ sFIDA uses a similar principle, where captured aggregates are labelled with fluorescent markers, and individual tau particles are analysed for fluorescence intensity, enabling the precise quantification of tau species, even at very low concentrations.

By enabling the visualisation of individual tau aggregates, SiMPull can provide insight into the heterogeneity of the tau aggregate population, making it possible to study distinct subpopulations, including specific post‐translationally modified or smaller and potentially more toxic aggregates that are difficult to detect using conventional methods (Scheme 5C). Furthermore, when combined with SMLM techniques, SiMPull offers the capability to determine the size and morphology of small tau aggregates, providing more detailed structural information at the nanoscale that is critical to both mechanistic studies and clinical applications. In addition, SiMPull may be modified to be combined with AFM, offering an atomic spatial resolution while maintaining the molecular specificity to tau aggregates.

However, SiMPull still faces challenges. It relies heavily on the quality and specificity of the antibodies used to capture tau species and, like other antibody‐based methods, can suffer from cross‐reactivity or incomplete capture of all relevant tau aggregates. Although SiMPull has been successfully applied to study complex biological samples, including brain tissue,^[32]^ serum,^[32]^ cell lysate^[178]^ and organoid media,^[179]^ challenges remain in optimising the technique to ensure the reliable detection of diverse tau species in these heterogeneous environments. The complexity of such samples, with the presence of other interacting proteins, adds a layer of difficulty in maintaining high sensitivity and specificity.

## 7 Antibody Specificity in Tau Research

Many of the methods discussed in this review, including immunostaining, ELISA, and SiMPull, rely on antibodies for the specific detection of tau species. These antibodies are critical for identifying and quantifying different forms of tau. However, the specificity and performance of antibodies can significantly impact the reliability and reproducibility of these methods.^[180]^

To address the heterogeneity of tau species, a wide range of antibodies has been designed to specifically detect tau, including total tau, phosphorylated tau, oligomeric tau, conformation‐specific tau, isoform‐specific tau and other post‐translationally modified tau.

A recent comprehensive evaluation of tau antibodies highlights the importance of validating antibody performance in detecting tau proteoforms.^[119]^ This study assessed 79 tau antibodies for Western blotting and 35 for immunohistochemistry, focusing on their specificity, sensitivity, and applicability in different biological contexts. Such validation efforts are crucial, as antibodies often show cross‐reactivity with other proteins, fail to distinguish between specific tau species or have binding affinities altered by PTMs.[^181^, ^182^] For example, although “total” tau antibodies are assumed to be unaffected by post‐translational modifications, phosphorylation has been shown to partially inhibit the binding of many such antibodies, including the widely used Tau‐5 clone.^[119]^

A range of antibodies specific to tau oligomers has been developed, such as TOMA and TOC1, have shown promise in recognising conformationally distinct toxic tau species.[^183^, ^184^] However, their specificity is limited by the structural heterogeneity of tau oligomers. As such, for example the “oligomer‐specific” T22 antibody also detected monomeric tau on Western blots.^[119]^ Structural diversity and overlapping epitopes among tau aggregates complicate antibody design, often leading to cross‐reactivity with non‐oligomeric tau forms, such as protofibrils or monomers. Additionally, epitope masking, particularly in β‐sheet‐rich or post‐translationally modified oligomers, can reduce detection sensitivity.^[118]^

The limitations of antibody‐based methods are particularly apparent in complex biological samples, where low‐abundance species such as tau oligomers are difficult to detect without significant background interference. The quality of antibodies also varies between commercial sources, necessitating rigorous validation for each experimental application.^[185]^ Furthermore, even high‐performing antibodies may have reduced efficacy in detecting tau at lower, endogenous levels.^[119]^

In conclusion, while antibody‐based techniques remain invaluable for studying tau aggregation, their reliability hinges on the specificity and sensitivity of the antibodies employed. Comprehensive validation, as demonstrated in recent studies, is essential for ensuring the reproducibility and accuracy of tau research. Moreover, integrating antibody‐based methods with complementary techniques, such as mass spectrometry, can provide more robust and comprehensive insights, helping to address the limitations of individual approaches. Future advances in antibody engineering and validation protocols will likely play an important role in overcoming current limitations and enabling more precise detection of disease‐relevant tau species.

## 8 Summary and Outlook

Tau protein plays a central role in maintaining cellular integrity and axonal transport, but its aggregation into oligomers and fibrils contributes to the onset and progression of tauopathies, including Alzheimer's disease and frontotemporal dementia. While much progress has been made in elucidating the structural properties of tau and its role in neurodegenerative diseases, significant gaps remain, particularly in the detection of early‐stage tau aggregates. These small tau oligomers are increasingly recognised as key drivers of neurotoxicity; however, they are not yet well understood due to certain limitations in both *in vitro* and *in vivo* studies. Furthermore, current detection methods struggle to capture these critical species due to a lack of sensitivity and/or specificity.

The ability to detect and characterise these small, early‐stage aggregates is essential not only for understanding the progression of tauopathies but also for developing early diagnostic tools. Current bulk methods fail to isolate these small, tau species, which are often the most toxic and biologically active. Single‐molecule techniques such as SiMPull offer a promising solution by enabling researchers to study tau aggregates at the single‐molecule level. This method can reveal critical information about the early conformational changes that precede fibril formation, potentially identifying tau oligomers that drive early disease progression. It is worth mentioning that some single‐molecule methods, such as AFM and cryoEM, offer an atomic resolution but lack molecular specificity, while other methods (e.g., SiMPull) rely on the specificity and affinity of the antibodies employed.

By enabling the early detection of toxic tau species, single‐molecule techniques hold significant potential for advancing both research and clinical practice. Early detection would allow for timely therapeutic intervention, potentially halting or slowing disease progression before substantial neuronal damage occurs. Furthermore, targeting these early‐stage oligomers could lead to the development of treatments aimed at preventing tau from reaching a fibrillar, aggregated state, offering new avenues for therapeutic intervention.

## Conflict of Interests

The authors declare no conflict of interest.

## Biographical Information

*David Klenerman is a physical chemist who graduated and completed his doctorate at Cambridge University working with Professor Ian Smith on infra‐red chemiluminescence for his PhD in 1985. This was followed by postdoctoral research at Stanford University, California with Professor Dick Zare on high overtone chemistry. He then returned to the U.K. and worked for seven years for BP Research in their Laser Spectroscopy Group before returning to the Department of Chemistry, University of Cambridge, progressing to a Professorship. He is currently a Royal Society GSK Professor of Molecular Medicine. At Cambridge his work has focussed on the development and application of physical methods, particularly laser spectroscopy and single molecule fluorescence, to biological and biomedical problems. He is a Fellow of the Royal Society and the Academy of Medical Sciences*.

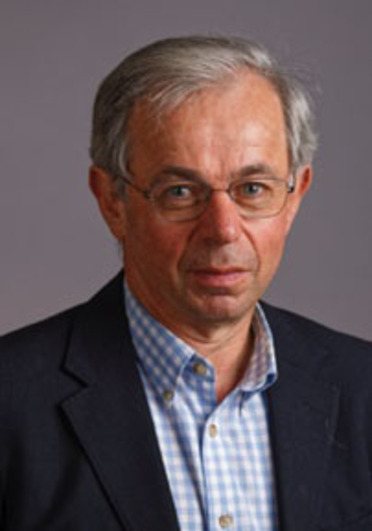

## Biographical Information

*Dorothea Böken is a PhD student at the University of Cambridge and the UK Dementia Research Institute, working with Professor Sir David Klenerman on developing single‐molecule methods, such as MAPTau, to study tau aggregates in complex biological samples. She completed her master's in Biological Chemistry at ETH Zurich, working with Professor Renato Zenobi on native mass spectrometry of protein‐DNA complexes. Dorothea also conducted research at ENS Paris with Professor Arnaud Gautier, developing novel chemogenetic reporters. Her PhD work focuses on applying novel techniques to better understand the molecular mechanisms of Alzheimer's disease and other tauopathies*.